# In Situ Generation of Fouling Resistant Ag/Pd Modified PES Membranes for Treatment of Pharmaceutical Wastewater

**DOI:** 10.3390/membranes12080762

**Published:** 2022-08-03

**Authors:** Rapelang Patala, Oranso T. Mahlangu, Hlengilizwe Nyoni, Bhekie B. Mamba, Alex T. Kuvarega

**Affiliations:** Institute for Nanotechnology and Water Sustainability, College of Science, Engineering and Technology, University of South Africa, Florida Science Campus, Johannesburg 1709, South Africa; 61051500@mylife.unisa.ac.za (R.P.); nyonih@unisa.ac.za (H.N.); mambabb@unisa.ac.za (B.B.M.)

**Keywords:** bimetallic nanoparticles, emerging organic compounds, polyethersulfone, antifouling properties, wastewater treatment

## Abstract

In this study, Ag and Pd bimetallic nanoparticles were generated in situ in polyethersulfone (PES) dope solutions, and membranes were fabricated through a phase inversion method. The membranes were characterized for various physical and chemical properties using techniques such as FTIR, SEM, AFM, TEM, EDS, and contact angle measurements. The membranes were then evaluated for their efficiency in rejecting EOCs and resistance to protein fouling. TEM micrographs showed uniform distribution of Ag/Pd nanoparticles within the PES matrix, while SEM images showed uniform, fingerlike structures that were not affected by the presence of embedded nanoparticles. The presence of Ag/Pd nanoparticles resulted in rougher membranes. There was an increase in membrane hydrophilicity with increasing nanoparticles loading, which resulted in improved pure water permeability (37–135 Lm^2^h^−1^bar^−1^). The membranes exhibited poor salt rejection (<15%), making them less susceptible to flux decline due to concentration polarization. With a mean pore radius of 2.39–4.70 nm, the membranes effectively removed carbamazepine, caffeine, sulfamethoxazole, ibuprofen, and naproxen (up to 40%), with size exclusion being the major removal mechanism. Modifying the membranes with Ag/Pd nanoparticles improved their antifouling properties, making them a promising innovation for the treatment of pharmaceutical wastewater.

## 1. Introduction

In recent years, there has been an increasing number of reports on the detection of emerging organic compounds (EOCs) such as pharmaceuticals, hormones, personal care products, and plasticizers in different water sources [1,2,3]. The presence of EOCs in water is of ecotoxicological importance, as they are a health hazard to living organisms at elevated concentrations. At high concentrations, EOCs may damage the human liver and lead to other negative health effects such as dermal lesions, weight loss in infants, respiratory disorders, ocular signs, neurological disorders, damage to the endocrine system, immunodeficiency, and reproduction disorders [4]. Some EOCs are classified as endocrine-disrupting compounds (EDCs) that interfere with the chronology of hormonal signals, thereby changing the developmental course of cellular tissues, leading to irreversible character changes [5]. The occurrence of EOCs in water is not prioritized, partly due to the high cost of analyses and less stringent global water quality guidelines [6]. Therefore, the removal of EOCs from water is imperative. Various water treatment processes such as biological treatment, adsorption, electrocoagulation, microbial fuel cell, sorption by wetlands, photocatalysis, activated sludge, and ion exchange processes have been applied for the removal of EOCs from water [7,8,9,10,11,12,13,14,15,16]. However, these processes present challenges that include inefficiency, the need to perform a post-treatment step, the generation of secondary sludge, high operating costs, large space for setup, and huge chemical usage. Membrane filtration offers an alternative and more efficient water treatment process whereby water purification is carried out in a single step without the use of chemicals. Membrane separation processes (MSPs), such as ultrafiltration (UF), nanofiltration (NF), and reverse osmosis (RO), have been effectively applied for the rejection and removal of EOCs from water [17,18,19,20]. The most common rejection mechanisms are size exclusion, [21,22,23] membrane-solute charge interactions, [24,25] as well as membrane-solute non-electrostatic interactions [26,27,28], which are all dictated by the membrane as well as the solute properties. This therefore emphasizes the importance of optimizing the membrane properties (e.g., molecular weight cut-off (MWCO), zeta potential, surface roughness, and hydrophobicity) [25,29,30] as well as solute properties (e.g., solute size, charge, and hydrophobicity) [18,22,31], which are important factors controlling the rejection of EOCs by pressure-driven membrane processes.

Although MSPs have advantages over other water treatment processes, membrane filtration has inherent problems such as susceptibility to fouling in addition to high capital cost (CapEX) and operating cost (OpEX) due to the high energy requirements to drive the feed through the membrane. Recent studies have focused on the fabrication of energy-efficient membranes with antifouling properties, where nanomaterials like graphene oxide, zinc oxide, titanium dioxide, and many others have been incorporated into the membrane polymer matrix. For example, Kusworo et al. [32] prepared PSf-TiO_2_/GO nanohybrid membranes for the degradation of organic contaminants in natural rubber wastewater. The addition of the nanoparticles improved the membrane morphology, hydrophilicity, permeation performance, and mechanical properties. In another study, Rosman et al. [33] used an in situ precipitation method to synthesize a PVDF-ZnO/Ag_2_CO_3_/Ag_2_O nanocomposite membrane which was resistant to fouling compared to the pristine membrane, and up to 35% ibuprofen rejection was reported under visible light irradiation.

Although sufficient research has been conducted on membranes incorporated with nanoparticles, knowledge about the EOC rejection properties of these membranes is still lacking, even though for commercial membranes, the rejection mechanisms have been determined. In studies that have investigated the removal of EOCs by membranes incorporated with nanoparticles, the EOC rejection mechanisms were not fully demonstrated. Therefore, this work investigated the antifouling properties of polyethersulfone (PES) membranes modified with in situ generated Ag/Pd bimetallic nanoparticles, and their ability to remove EOCs from water, paying particular attention to the major solute rejection mechanisms. Ag and Pd nanoparticles were selected because at the nano range, these metals and many others have been reported to enhance membrane properties and performance, [22,34,35]. Bimetallic metal nanoparticles are important because their characteristics are often quite different from their monometallic counterparts. Further, bimetallic nanoparticles are particularly useful due to their flexibility, composition, and structure [36]. PES polymer shows interesting properties which include thermal stability, chemical resistance, and strength. These properties are retained when the polymer is transformed into a membrane. The fabricated membranes were characterized and evaluated for performance in a crossflow filtration system. The solute rejection properties were investigated using five model organic compounds of varying size, charge, and hydrophobicity. Finally, membrane antifouling studies were performed using bovine serum albumin (BSA) as a model foulant. BSA was chosen as model protein foulant because it represents a major group of organic foulants in membrane treatment (particularly for wastewater reclamation), and it has been widely used to study membrane fouling [37,38].

## 2. Materials and Methods

### 2.1. Materials

**Figure 1:**
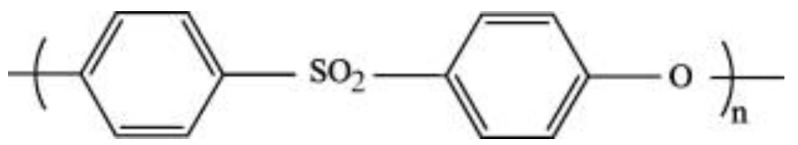
Chemical structure of polyethersulfone (PES) [39].

Polyethersulfone (PES) (Veradel^®^ 3000P, Mw = 62,000 g·mol^−1^) was supplied by Solvay Advanced Polymers (Brussels, Belgium) (Figure 1). Sodium borohydride (NaBH_4_), triethyl phosphate (TEP) ((C_2_H_5_)_3_PO_4_), palladium chloride (PdCl_2_) and silver acetate (AgC_2_H_3_O_2_) were purchased from Sigma-Aldrich, South Africa. All chemicals were of analytical grade and used as received.

### 2.2. Membrane Fabrication

A non-solvent induced phase separation method was used for the fabrication of the membranes, following a previously reported procedure [40,41]. A certain amount of PES granules was dissolved in TEP at 60 °C and stirred for 5 h until a homogeneous solution was formed. This was followed by dissolving the metal precursors (silver acetate and palladium chloride) in 5 mL TEP, which was then added to the previously prepared dope solution and the mixture was left to homogenize for 2 h. Thereafter, NaBH_4_ (0.5 M) was added dropwise until in excess to reduce the metal ions to bimetallic nanoparticles. The initial addition of few drops of NaBH_4_ into the reaction mixture resulted in a color change, i.e., from clear to yellow. As more drops of NaBH_4_ were added, a change in color from yellow to brown was observed; this indicated excess NaBH_4_. Different amounts of Ag and Pd precursor concentrations (1:1) were used (Table 1) to fabricate membranes with varying amounts of embedded bimetallic nanoparticles.

The casting solution was left to degas at room temperature overnight, after which the membranes were cast with a knife (250 μm air gap) on a glass plate and immersed in a deionized water coagulation bath maintained at 25 °C. The nascent membranes were peeled off from the glass plate and placed in a freshwater bath at room temperature (25 °C) to remove any residual solvent and additives. The membranes were then kept in de-ionized water for further use.

### 2.3. Membrane Characterization

#### 2.3.1. Scanning Electron Microscopy (SEM) and Energy Dispersive X-Ray Spectroscopy (EDS)

Both the surface morphologies and cross-sectional micrographs of the membranes were obtained using a scanning electron microscope (Jeol JSM IT300, Tokyo, Japan) with an attached EDS analyzer (EDS, Jeol JSM IT300, Tokyo, Japan). An irradiation beam of 15 kV was applied. Membranes were first dried in desiccators for at least 24 h and gold coated at a current of 25 µA for 50 s using an SCD 005 Cool Sputter Coater (BalTec, Lübeck, Germany). For cross-section analysis, the membranes were dipped into liquid nitrogen, and the frozen membranes were broken for edge analysis. These membranes were then placed on carbon tape and coated with gold before imaging.

#### 2.3.2. Atomic Force Microscopy (AFM)

A WITec Alpha 300 atomic force microscope or AFM (WITec, GmbH, Ulm, Germany) was used to obtain AFM surface images of the dried membranes. Measurements were conducted in semi-contact mode using reflex coated FM (AC), 2.8 N·m^−1^, 75 kHz AFM arrow cantilevers.

#### 2.3.3. Contact Angle (CA)

Water contact angle measurements were performed by putting a sessile water drop (5 μL) on the dry membrane surface. The results were recorded with a goniometer (DSA 10-MK2, Kruss, Kruss, Germany). The contact angle values were averaged from at least ten measurements from different locations on each membrane surface.

#### 2.3.4. Transmission Electron Microscopy (TEM)

A JEOL JEM-2100F at an accelerating voltage of 200 kV was used to obtain transmission electron microscopy (TEM) micrographs. The dry Ag/Pd PES membranes were broken into small pieces and dissolved in triethyl phosphate (TEP). A drop of the solution was then placed onto a copper grid for TEM analysis. ImageJ software was used to determine the average size of nanoparticles.

#### 2.3.5. Thermo-Gravimetric Analysis (TGA)

Thermographs of the PES and Ag/Pd PES membranes were obtained using a TGA analyzer (TGA 5500-0026, TA Instruments, Newcastle, DE, USA) TGA analysis was performed at a ramp rate of 10 °C·min^−1^ and N_2_ was used as the purge gas at a flow rate of 50 mL·min^−1^. The temperature was ramped from 30 °C to 900 °C.

#### 2.3.6. Fourier Transform Infrared Spectroscopy (FTIR)

Fourier transform infrared (FTIR) spectra of the prepared PES and Ag/Pd PES membranes were investigated using an FTIR frontier from Perkin Elmer. The analysis was carried out in the wavenumber range of 600–1800 cm^−1^_._

#### 2.3.7. Zeta Potential Measurements

The zeta potential of the membranes was quantified by measuring streaming potential of the membranes at pH 7 using a SurPASS Electrokinetic Analyzer (Anton Paar GmbH, Graz, Austria). The background electrolyte was 10 mM KCl (Sigma Aldrich, Johannesburg, South Africa). Measurements were done using the tangential mode of analysis at applied pressure of 250 mbar and gap height of 115 μm. The zeta potentials (ζ) were estimated based on the Helmholtz-Smoluchowski equation (Equation (1)); where Δ*V* (mV) is the measured streaming potential, *η* (Pas) is electrolyte viscosity, Δ*P* (Pa) is applied pressure, *δ* (µS·cm^−1^) is electrolyte conductivity and ε (C^2^N^−1^m^−2^) is permittivity of water.

(1) $$\zeta =\frac{∆V\eta \delta }{∆P\varepsilon }$$

#### 2.3.8. Measurement of Porosity and Mean Pore Radius

The dry-wet weight approach was used to predict the porosity of the synthesized membranes, where small portions of membranes were kept in water for at least 12 h and the weight of the wet membranes was measured using an analytical balance after wiping off superficial water on the membrane surfaces. This was followed by drying the wet membranes in an oven at 45 °C for 24 h. Thereafter, the weight of the dry membranes was measured. Membrane porosity (ε) was then calculated from the weight of the wet membrane (*W_w_*, g), the weight of the dry membrane (*W_d_*, g), the membrane area (*A*, cm^2^), membrane thickness (*δ,* cm) (measured using a digital micrometer) and density of water (*ρw,* g·cm^−3^) according to Equation (2):

(2) $$\varepsilon \left(\%\right)=\frac{W_{w}-W_{d}}{\rho_{w}A\delta }\times 100$$

The mean pore radius was then predicted utilizing the Guerout–Elford–Ferry equation (Equation (3))

(3) $$r_{m}=\frac{\sqrt{\left(2.9-1.75\varepsilon \right)8ŋ\delta Q}}{\varepsilon \cdot A\cdot ∆P}$$

where *r_m_* is the mean pore radius (m), *ε* is the membrane porosity, ŋ is the water viscosity (8.9 × 10^−4^ Pa·s), *δ* is the membrane thickness (m), *Q* is the volume of permeated water per unit time (m^3^s^−1^), A is the effective area of the membrane (m^2^), and Δ*P* is the applied pressure (Pa).

### 2.4. Filtration Experiments and EOC Rejection

#### 2.4.1. Filtration Setup

**Figure 2:**
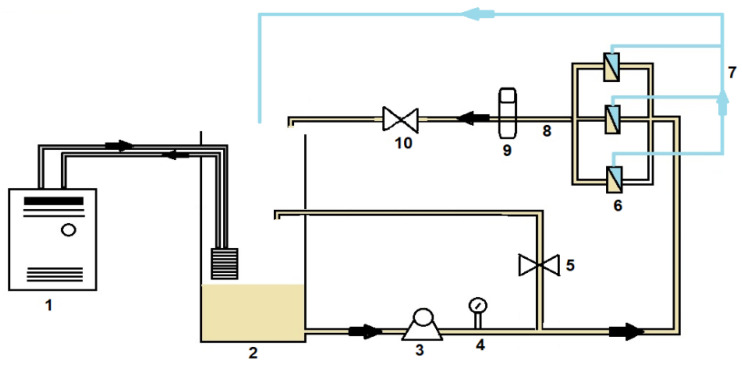
Crossflow filtration setup: 1—chiller; 2—reservoir; 3—high pressure pump; 4—pressure gauge; 5—by-pass valve; 6—membrane cell; 7—permeate; 8—concentrate; 9—flow meter; 10—concentrate valve.

The membrane filtration properties were investigated using a crossflow filtration setup (Figure 2) with the following channel dimensions: channel width of 3.5 cm, channel length of 8.5 cm, and channel height of 0.1 cm. The feed solution was contained in a stainless-steel tank (20 L) and was pumped through the membrane cells by a high-pressure pump (Hydra-Cell; Wanner Engineering, Minneapolis, Minnesota). To eliminate the influence of varying permeate flux on solute rejection, an initial permeate flux of 60 L·m^−2^h^−1^ was used for all filtration experiments. This was achieved by varying the applied pressure to obtain the desired flux based on the pure water permeability of the different membranes. A crossflow velocity of 0.2 m·s^−1^ was maintained. Filtration experiments were performed in recycling mode, where both the retentate and permeate were recycled back into the feed tank. Prior to conducting any filtration experiments, the membranes were cleaned with milli-Q water and compacted at 600 kPa until the flux was stable. This was followed by conducting pure water flux experiments at varying applied pressures (from 100 kPa to 600 kPa). Thereafter, the rejection of 2000 g·L^−1^ MgSO_4_, 5.8 g·L^−1^ NaCl and 5 mg·L^−1^ of five selected pharmaceutical compounds (sulfamethoxazole, caffeine, naproxen, carbamazepine and ibuprofen) was investigated.

Membrane water flux ($J_{w}$) was estimated from the volume of water collected (V, L) at specific time (t, s) based on Equation (4); where A is the membrane area (m^2^).

(4) $$J_{w}=\frac{V}{At}$$

Solute rejection (R%) was based on the concentration of solute in the permeate ($C_{p}$) and feed ($C_{f}$) (Equation (5)).

(5) $$R\left(\%\right)=\left(1-\frac{C_{p}}{C_{f}}\;\right)\times 100$$

The concentration of salts in both the feed and permeate was measured using an electrical conductivity meter (Consort C6010 conductivity meter, Consort, Turnhout, Belgium) while the concentration of pharmaceuticals was measured as total organic carbon (TOC) using a TOC analyzer (TOC Fusion, Teledyne Tekmar, Mason, OH, USA) with a limit of detection of 0.1 mg·L^−1^.

#### 2.4.2. Selection of EOCs and Analysis

Five pharmaceutical compounds were selected based on their size, hydrophobicity, and charge (Table 2). The solutes were individually dosed at concentrations of 5 mg·L^−1^. The pharmaceuticals were dosed at high concentrations in order to permit solute quantification in the permeate at 98% rejection. Rejection of the organics was investigated at a permeate flux of 60 L·m^−2^, neutral pH, and 10 mM NaCl background electrolyte concentration. Solute rejection was determined after 4 h of equilibration in order to eliminate the influence of temporal adsorption of solutes on the membrane surface on solute rejection.

### 2.5. Fouling Experiments

Fouling experiments were conducted in a crossflow filtration set-up (Figure 2) where the membranes were individually fouled with 20 mg·L^−1^ bovine serum albumin (BSA) at 25 °C. The background electrolyte solution was maintained at 10 mM using NaCl. The BSA used in this study had a molecular weight of 66,430 g·mol^−1^ and negative zeta potential of −15.8 mV [42]. All experiments were started at initial flux of 60 L·m^−2^h^−1^ and crossflow velocity of 0.2 m·s^−1^. This was to ensure that the fouling propensity of the membranes was not misrepresented since fouling also depends on the initial flux. The membranes were allowed to foul for 72 h and the flux was measured at predetermined times. The most important parameters for the determination of antifouling properties of membranes are the flux recovery ratio (FRR), the total flux decline ratio ($R_{t}$), as well as irreversible ($R_{ir}$) and reversible ($R_{r}$) fouling ratios. To determine the fouling resistance properties, the membranes were washed with milli-Q water after the 72 h of fouling experiments, and the pure water fluxes ($J_{w2}$) were re-determined. The different fouling ratios were were then quantified using Equations (6)–(9).

(6) $$FRR\;\left(\%\right)=\left[\frac{J_{w2}}{J_{w1}}\right]\cdot 100$$

(7) $$R_{t}\left(\%\right)=\left[1-\frac{J_{f}}{J_{w1}}\right]\cdot 100$$

(8) $$R_{r}\left(\%\right)=\left[\frac{J_{w2}-J_{f}}{J_{w1}}\right]\cdot 100$$

(9) $$R_{ir}\left(\%\right)=\left[\frac{J_{w1}-J_{w2}}{J_{w1}}\right]\cdot 100$$

where ($J_{w1}$) is pure water flux prior to fouling, ($J_{w2}$) is pure water flux after fouling, and ($J_{f}$) is flux of the foulant solution.

### 2.6. Investigation of Nanoparticle Leaching

Ag and Pd are heavy metals whose presence in water would be harmful to the environment, even at low concentrations. Therefore, the leaching of the nanoparticles was investigated. Briefly, new membrane coupons were compacted at 600 kPa until the flux was stable. This was followed by filtration of ultrapure water over a period of 12 h, where samples of the permeate, concentrate, and feed were collected and analyzed for the presence of Ag and Pd using inductive coupled plasma mass spectroscopy (ICP-MS) under carefully controlled conditions.

## 3. Results and Discussion

### 3.1. Membrane Characteristics

#### 3.1.1. FTIR

**Figure 3:**
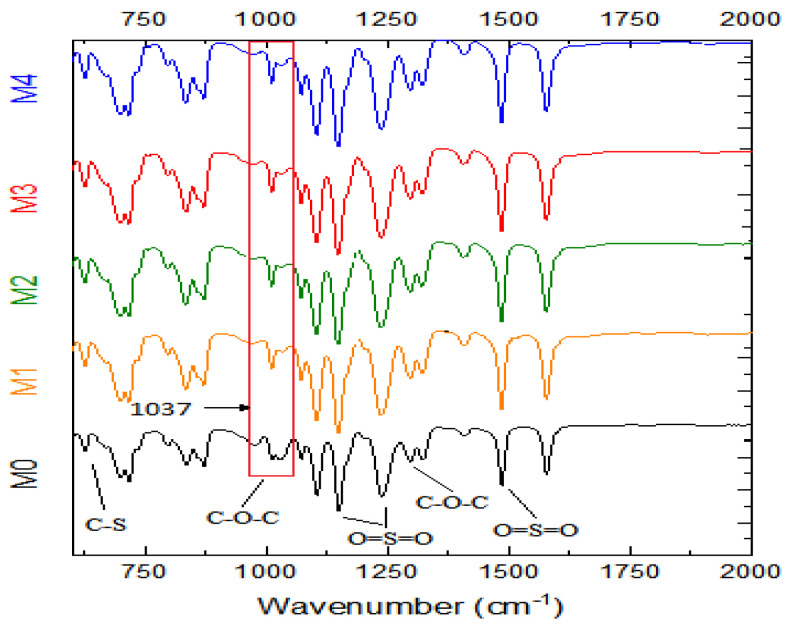
FTIR spectra of Ag/Pd PES membranes: M0 − PES + 0% Ag/Pd, M1 − PES + 0.1% Ag/Pd, M2 − PES + 0.15 Ag/Pd, M3 − PES + 0.2% Ag/Pd and M4 − PES + 0.25% Ag/Pd nanoparticles.

Figure 3 shows the FTIR spectra of Ag/Pd PES membranes. Even after in situ generation of AgPd bimetallic nanoparticles, all the PES characteristic peaks remained virtually unaltered in all the membranes confirming the structural integrity of the membrane even after embedding Ag/Pd nanoparticles. The C-stretching and C=C stretching on the aromatic rings were identified with peaks at 620 and 880 cm^−1^, respectively. The sulfonyl group which is characteristic of PES was confirmed with peaks at 1150 cm^−1^, 1240 cm^−1^ and 1481 cm^−1^. The aromatic ether (C–O–C) group was assigned to the peak at 1244 cm^−1^. These results were in agreement with previous reports [43]. However, at 1037 cm^−1^ the aromatic ester functional group appeared more conspicuous for the membranes loaded with Ag/Pd nanoparticles. This peak was also observed by other researchers and ascribed to the presence of the Ag nanoparticles [44,45].

#### 3.1.2. TGA

**Figure 4:**
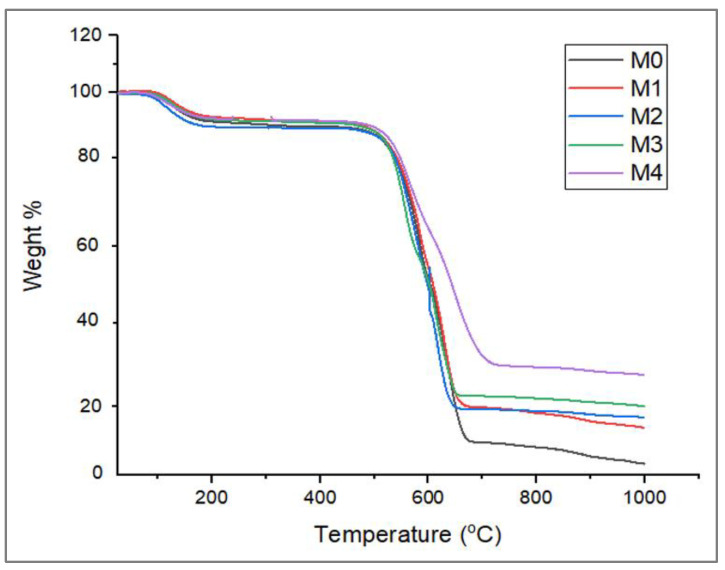
TGA thermograms of Ag/Pd PES membranes: M0 − PES + 0% Ag/Pd, M1 − PES + 0.1% Ag/Pd, M2 − PES + 0.15 Ag/Pd, M3 − PES + 0.2% Ag/Pd and M4 − PES + 0.25% Ag/Pd nanoparticles.

Figure 4 shows thermograms of both pristine PES and Ag/Pd modified PES membranes. Pristine PES membranes are reported to decompose at around 500 °C [46]. The presence of Ag and Pd nanoparticles did not alter the structural integrity of the polymer, as there were no remarkable shifts in the decomposition temperature. The negligible effects of the nanoparticles on the thermal stability of the membranes was due to the small amounts of the nanoparticles entrapped in the membranes (up to 0.25 wt%).

#### 3.1.3. SEM and EDS

**Figure 5:**
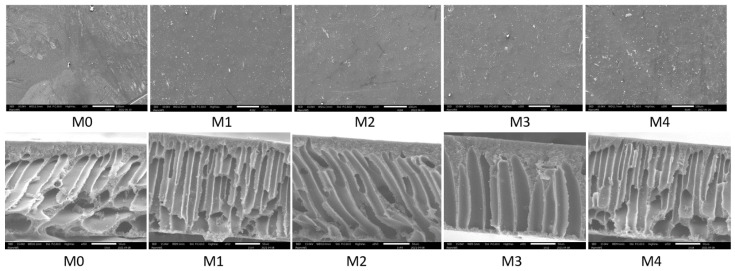
Surface (**Top**, scale bar: 100 µm) and cross-sectional (**Bottom**, scale bar: 50 µm) SEM micrographs of the membranes: M0 − PES + 0% Ag/Pd, M1 − PES + 0.1% Ag/Pd, M2 − PES + 0.15 Ag/Pd, M3 − PES + 0.2% Ag/Pd and M4 − PES + 0.25% Ag/Pd nanoparticles.

Figure 5 shows surface and cross-sectional micrographs of the pristine and the Ag/Pd PES membranes. The surface of the membranes appeared smooth and the addition of Ag/Pd nanoparticles did not alter the surface morphology. The membranes are characterized by a thin active layer and long, fingerlike microvoids as internal structures. The skin layer showed an asymmetric sponge structure on top of several arranged channels forming the bulk of the membrane cross-section. These membrane structures have been reported to result in higher water fluxes [47]. The incorporation of Ag/Pd nanoparticles resulted in increase in the size of the microvoids. Not only are the microvoids enlarged for nanoparticle loaded membranes, but the channels are also more ordered as opposed to pristine membrane (M0). Enlarged microvoids allow easy passage of water leading to higher fluxes, but they may also allow for passage of smaller solutes like salts leading to low rejections. However, rejection (especially rejection of organic compounds) is not entirely through size exclusion but also through electrostatic interactions as well as non-electrostatic interactions such as hydrophobic interactions [26].

**Figure 6:**
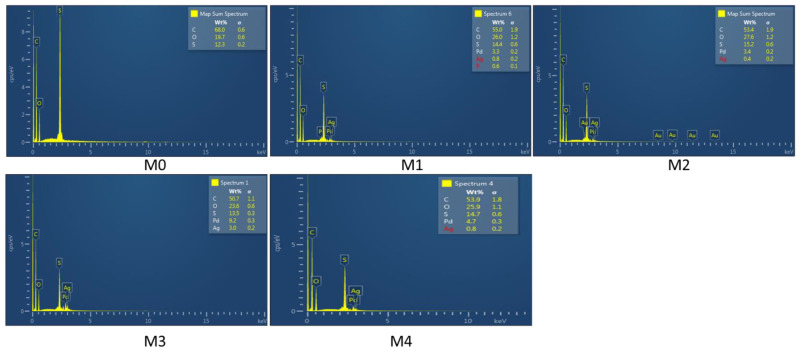
EDS spectra of Ag/Pd PES membranes: M0 − PES + 0% Ag/Pd, M1 − PES + 0.1% Ag/Pd, M2 − PES + 0.15 Ag/Pd, M3 − PES + with 0.2% Ag/Pd and M4 − PES + 0.25% Ag/Pd nanoparticles.

The presence of Ag and Pd in the membrane polymer matrix was confirmed by EDS analysis, where Ag and Pd signature peaks were observed (Figure 6). In the spectra C, O, and S were also observed; these were attributed to the PES polymer. The low intensities of the Ag and Pd peaks can be attributed to the low concentrations of the metal precursors used during the membrane fabrication process. An increase in the peak intensities of Ag and Pd was noted from M0 to M4. These results confirmed the successful entrapment of the bimetallic nanoparticles in the membranes.

#### 3.1.4. TEM Micrographs

**Figure 7:**
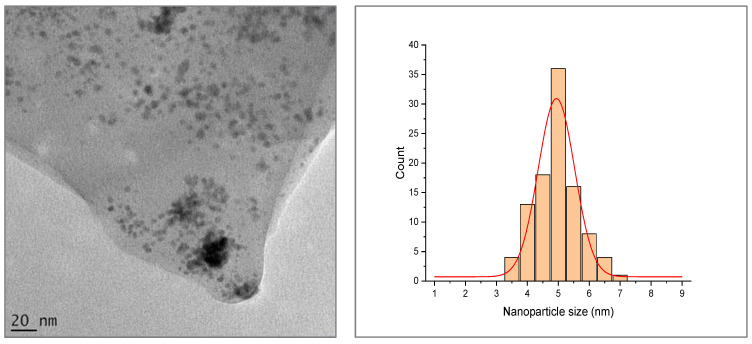
TEM micrograph for Ag/Pd nanoparticles and their size distribution.

Figure 7 shows the uniform size and narrow size distribution of the AgPd bimetallic nanoparticles. The nanoparticle showed an average particle size of 4.71 ± 0.42 nm. The AgPd bimetallic nanoparticles were spherical in shape and uniformly dispersed. This is an important parameter with regard to achieving a high surface area in membrane applications.

#### 3.1.5. AFM Micrographs and Surface Roughness

**Figure 8:**
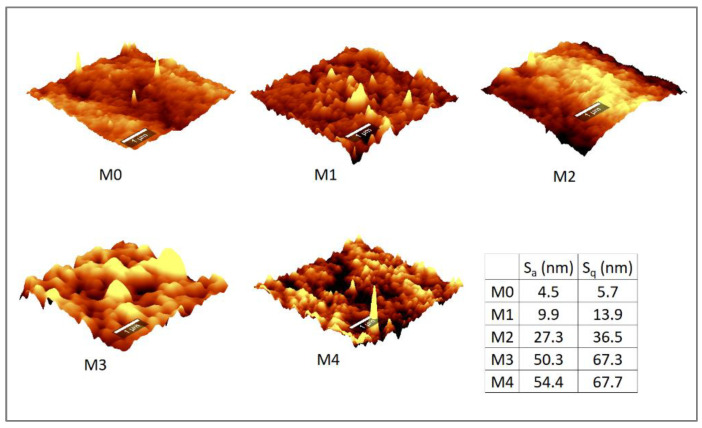
Atomic force micrographs of PES membranes with and without Ag/Pd nanoparticles: M0 − PES + 0% Ag/Pd, M1 − PES + 0.1% Ag/Pd, M2 − PES + 0.15 Ag/Pd, M3 − PES + 0.2% Ag/Pd and M4 − PES + 0.25% Ag/Pd nanoparticles.

Membrane roughness increased with the increase in the amount of embedded Ag/Pd nanoparticles (Figure 8) as increases in both the average arithmetic roughness (S_a_) and the root mean square roughness (S_q_) values were observed. Membrane surface roughness increased from S_a_ = 4.5 nm and S_q_ = 5.7 nm for M0 to S_a_ = 54.4 nm and S_q_ = 69.7 nm for M4. The increasing trend in membrane roughness with increasing amount of embedded nanoparticles is attributed to the increase in membrane heterogeneity due to the presence of the additives [22]. Further, the entrapment of Ag/Pd nanoparticles into the PES polymer might have initiated a polymerization reaction that caused the agglomeration of the nanoparticles located closer to the surface leading to a change in the height of the valley and ridges on the membrane surface. This may result in increased valleys, thus the observed increase in roughness of the membrane surface [35].

Contradictory observations have been reported regarding the relation of membrane surface roughness to fouling. Some authors have noted that membranes with high surface roughness are more prone to fouling compared to membranes with smoother surfaces, [48] while other researchers claimed no remarkable effect of membrane surface roughness on membrane fouling [49]. The differences in observations could be due to membrane fouling not being entirely controlled by membrane roughness, but also by other parameters, such as membrane zeta potential and hydrophobicity, as well as the operating conditions [50].

#### 3.1.6. Membrane Hydrophobicity, Fluxes, Porosity, Zeta Potential, Mean Pore Radius, and Salt Rejection Properties

Table 3 shows water contact angles, fluxes, porosity, zeta potential, mean pore radius, and salt rejection properties of the Ag/Pd PES membranes. The PES membranes were negatively charged at neutral pH due to the dissociation of sulfonic groups. The addition of Ag/Pd nanoparticles lowered the zeta potential from −42.48 mV to −31.29 mV. The membranes became more hydrophilic with increase in Ag/Pd nanoparticles as shown by the decrease in water contact angle. This observation concurs with literature reports and is attributed to the improvement in water uptake by the membranes due to the presence of nanoparticle additives [22,32]. Improvement in water uptake was also demonstrated by increase in membrane bulk porosity with addition of nanoparticles. The membranes were fabricated for the treatment of pharmaceutical wastewater where hydrophilic membranes are desirable for higher fluxes and reduced membrane-solute hydrophobic interactions leading to higher rejections [22]. Additionally, wastewater often has high concentrations of natural organic matter (NOM) which has the potential to foul hydrophobic membranes thereby changing the membrane surface properties and filtration properties [51].

Based on the water contact angles, improved porosity and surface roughness, it was postulated that membranes with embedded Ag/Pd (M1–M4) will have higher permeability to pure water compared to the pristine membrane (M0). This is because membranes M1–M4 had lower contact angles and larger pores compared to M0. The contact angle decreased from 72.6° to 51.3° while the mean pore radius increased from 2.39–4.70 nm with increase in Ag/Pd concentration from 0 to 0.25% (Table 1). Flux measurements showed increasing permeability for bimetallic nanoparticle loaded membranes. Water permeability increased with increasing concentration of nanoparticle loading which was consistent with contact angle measurements.

Although the membranes were highly permeable to pure water, the rejection of salts (NaCl and MgSO_4_) was very poor; this is a known characteristic of PES membranes. However, membranes with excellent salt rejection properties are not always required, especially in wastewater reclamation, where the feed water has low salt concentrations. It is also expected that membranes with poor salt rejection properties will be more energy efficient, as the effects of concentration polarization will be minimum.

### 3.2. Trace Organic Compounds Rejection Properties

**Figure 9:**
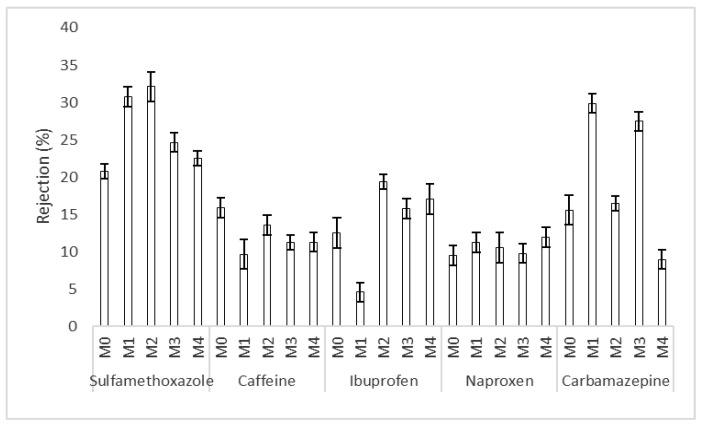
Rejection of organic compounds by fouled and virgin membranes: M0 − PES + 0% Ag/Pd, M1 − PES + 0.1% Ag/Pd, M2 − PES + 0.15 Ag/Pd, M3 − PES + 0.2% Ag/Pd and M4 − PES + 0.25% Ag/Pd nanoparticles.

Figure 9 shows the rejection of organic compounds by the Ag/Pd PES membranes. It was noted that in most instances, the addition of Ag/Pd improved the rejection of organic compounds as the rejection of organic compounds by Ag/Pd PES membranes was higher than that with the pristine membrane. However, this was exceptional for caffeine rejection, where the presence of Ag/Pd nanoparticles did not improve its rejection. The improvement in rejection of organic compounds by membranes modified with Ag/Pd could have been due to a reduction in membrane-solute affinity interactions. Membrane modification with Ag/Pd nanoparticles improved membrane hydrophilicity, thus reducing membrane-solute hydrophobic interactions, resulting in higher rejection [22]. There was no evidence of concentration polarization that may have influenced solute rejection, as there was no noticeable increase in the trans-membrane pressure (TMP) over time [52]. The negligible influence of concentration polarization effects could be linked to the poor membrane salt rejection properties.

### 3.3. Solute Rejection Mechanisms by the Ag/Pd PES Membranes

**Figure 10:**
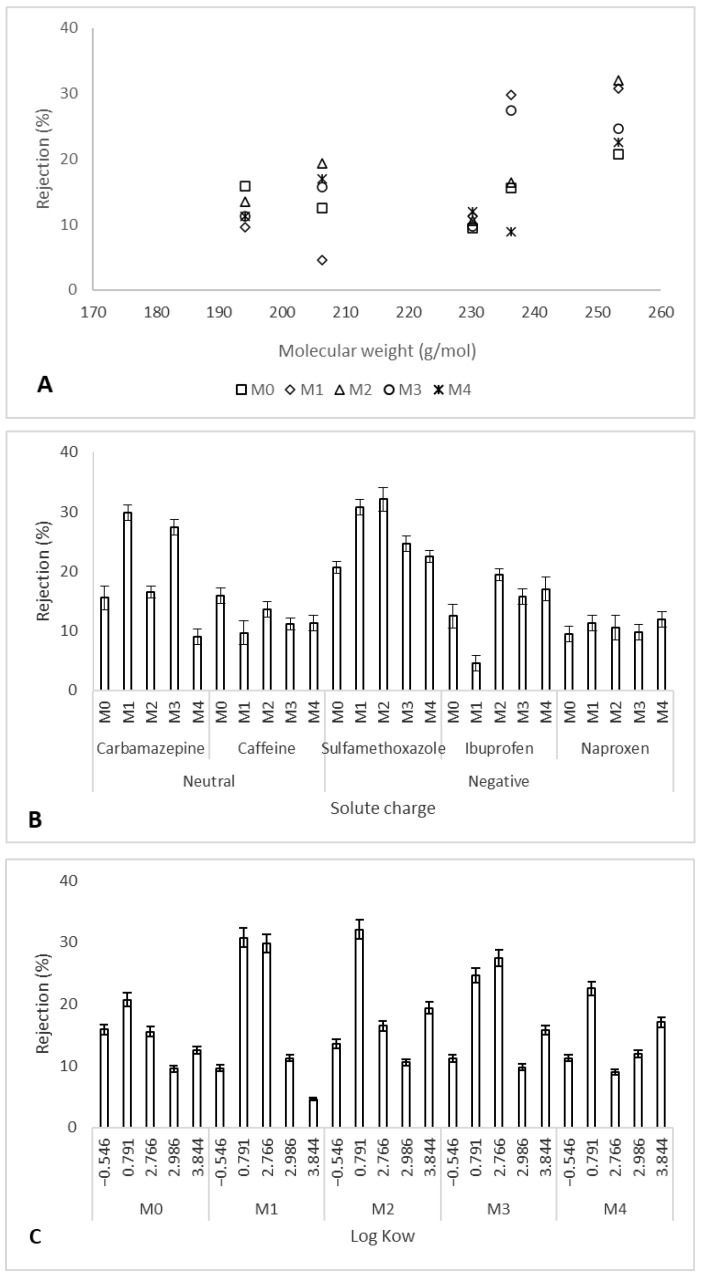
Rejection of the organic solutes as a function of molecular weight (**A**); solute charge (**B**) and solute hydrophobicity (**C**): M0 − PES + 0% Ag/Pd, M1 − PES + 0.1% Ag/Pd, M2 − PES + 0.15 Ag/Pd, M3 − PES + 0.2% Ag/Pd and M4 − PES + 0.25% Ag/Pd nanoparticles.

Previous studies have shown that the rejection of organic compounds is via sieving effects, where the solutes are rejected based on their molecular weight [21,23]. This results in larger solutes showing higher rejection rates compared to smaller solutes which can easily permeate through the membrane. To investigate the role of sieving effects for the PES and Ag/Pd PES membranes, rejection was plotted as a function of solute molecular weight (Figure 10A). The rejection of the selected organic compounds increased with the molecular weight of the solutes, thus confirming that solute rejection was mainly through sieving effects and the results were in agreement with previous reports [22,25]. However, there were some deviations where some larger solutes were lowly rejected showing that rejection was not entirely through size exclusion but also through other mechanisms. These mechanisms include membrane-solute charge interactions [24,25] and membrane-solute non-electrostatic or hydrophobic interactions [26,27,28].

To investigate the role of membrane-solute charge interactions on rejection, solute removal was plotted as a function of solute charge (Figure 10B). The model solutes represented neutral as well as negatively charged compounds. The influence of charge interactions played no major role in solute rejection, as both charged and uncharged solutes were rejected to more or less the same extent. Regarding neutral compounds, carbamazepine was generally rejected more than caffeine. Both solutes were neutral at the working pH of approximately 7. However, carbamazepine has a slightly bigger molecular size than caffeine. Therefore, carbamazepine was rejected more than caffeine through size exclusion. Regarding negatively charged solutes, sulfamethoxazole > naproxen > ibuprofen in terms of size and the rejection order followed the size or molecular weight with sulfamethoxazole being highly rejected while ibuprofen was least rejected. The absence of no obvious trend of solute rejection based on charge does not imply that charge interactions were not important in solute rejection. Previous studies have already shown the dependency of solute rejection on charge interactions [24,25]. For the Ag/Pd PES membranes, the role of charge interactions on rejection were overshadowed by sieving effects which were more apparent as shown in Figure 10A.

It has been shown that the rejection of organic compounds is not only influenced by molecular size of the solute and charge, but also non-electrostatic or affinity interactions. Affinity interactions comprise hydrophobic interactions, Van der Waals interactions, hydrogen bonding and dielectric effects. The concept of membrane-solute hydrophobic interactions has shown that hydrophilic solutes are often rejected more by hydrophobic membranes compared to the rejection of hydrophobic organics by hydrophobic membranes [26,27,28]. Therefore, the rejection of organic compounds was plotted as a function of solutes hydrophobicity (Figure 10C). A closer look at each membrane (except for M4) shows that more hydrophilic solutes were rejected slightly higher than hydrophobic compounds. This was exception for caffeine which showed low rejections despite the smaller Log K_ow_. However, this made sense because caffeine was the smallest molecule, and thus, it easily permeated through the membranes with minimal sieving effects. Sulfamethoxazole being hydrophilic and having a larger molecular weight (253.278 g/mol) was highly rejected by all the membranes. These results imply that non-electrostatic interactions or hydrophobic interactions played a role in the rejection of the model compounds in addition to size exclusion effects.

In general, the synthesized Ag/Pd PES membranes showed the potential to reject organic compounds from contaminated water sources. The major rejection mechanism was through size exclusion (where larger solutes were rejected slightly more than smaller solutes) and hydrophobic interactions. More studies are required to determine the role of interaction energies between the membranes and the solutes in relation to solute rejection [22].

### 3.4. Membrane Resistance to Protein Fouling

**Figure 11:**
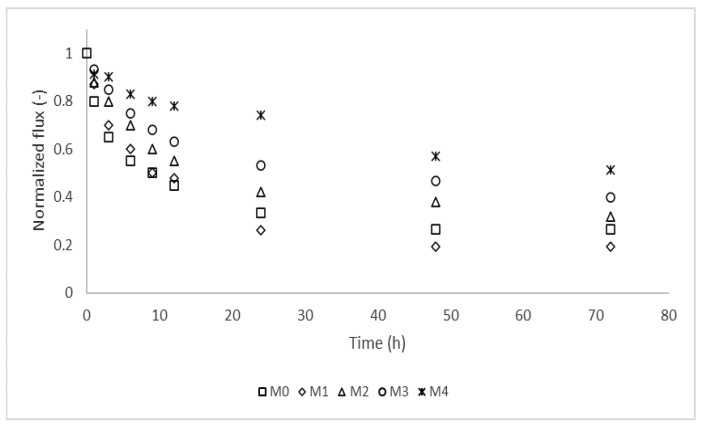
Membrane flux decline due to fouling by 20 mg/L bovine serum albumin (BSA): M0 − PES + 0% Ag/Pd, M1 − PES + 0.1% Ag/Pd, M2 − PES + 0.15 Ag/Pd, M3 − PES + 0.2% Ag/Pd and M4 − PES + 0.25% Ag/Pd nanoparticles.

Fouling experiments showed that the pristine membrane suffered more flux decline due to fouling by protein BSA (Figure 11). The membrane fouling resistance increased with increasing nanoparticle loading. This can be attributed to the increasing hydrophilicity of the membrane with increasing Ag/Pd loading. A decrease in membrane fouling due to the addition of nanoparticles has been observed before; this was attributed to the absence of extensive membrane-foulant hydrophobic interactions [32,33]. There was a steep decline in flux in the initial stages of membrane fouling and then the flux declined gradually and stabilized after about 48 h of fouling. The high fouling rate within the initial filtration stages can be attributed to the availability of more foulants to interact with the clean membrane surface (by convective transport and permeation drag) leading to higher fouling propensity [50]. This leads to decrease in foulant concentration in the feed and therefore a decreased fouling rate afterwards. Finally, depletion of foulants in the feed tank results in a steady flux over time. The depletion of foulants was confirmed using a turbidity meter where the turbidity of the feed decreased substantially from 20 ± 2.3 NTU to 0.5 ± 0.2 NTU (Eutech TN-100 turbidimeter, Thermo Fisher Scientific, Waltham, MA, USA). Previous studies have shown the dependency of membrane fouling on the concentration of foulants. Basically, membranes fouled more at higher foulant concentrations due to the formation of a dense fouling layer [50]. In this study, the foulant concentration used was higher than could be expected in real water samples. Therefore, in reality, the membranes are assumed to show less fouling propensity than reported in this work.

A visual inspection of the membranes showed that the pristine membrane had more foulant deposited on the surface compared to the membranes with nanoparticles, while the 0.25% Ag/Pd PES membrane had the least deposited foulant. It is believed that the deposited fouling layers reduced permeate flux by providing additional hydraulic resistance to water flow.

**Figure 12:**
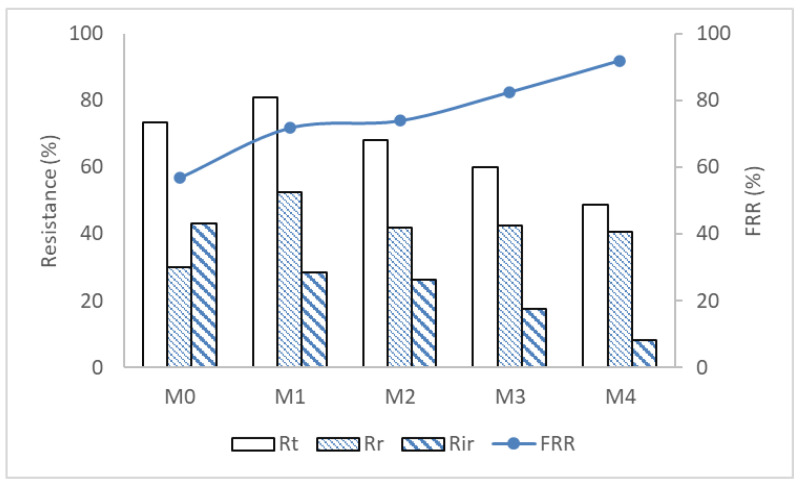
Fouling parameters of the different PES membranes: M0 − PES + 0% Ag/Pd, M1 − PES + 0.1% Ag/Pd, M2 − PES + 0.15 Ag/Pd, M3 − PES + 0.2% Ag/Pd and M4 − PES + 0.25% Ag/Pd nanoparticles.

Membrane fouling falls into two categories, namely, reversible fouling (*R_r_*) and irreversible fouling (*R_ir_*). In reversible fouling, the permeate flux can be restored by hydraulic cleaning of the fouled membrane, whereas chemical cleaning is required when fouling is irreversible. Total fouling (*R_t_*) is thus a combination of *R_r_* and *R_ir_*. To understand antifouling properties of the pristine and modified membranes, *R_t_*, *R_r_* and *R_ir_* were quantified. In addition, the flux recovery ratio (*FRR*) was also quantified. Membranes incorporated with Ag/Pd nanoparticles had higher *FRR* than the pristine membrane, showing superior fouling resistance (Figure 12). For the pristine membrane (M0), flux decline was mainly due to irreversible fouling which contributed more to the total fouling and the lowest *FRR*. Upon addition on Ag/Pd nanoparticles, irreversible fouling was reduced, thus allowing for higher flux recovery from simply washing the membranes with water. The *FRR* increased from 56.8% (M0) to 91.9% (M4). The improvement in fouling resistance could be attributed to the improvement in hydrophilicity of the membranes upon addition of Ag/Pd nanoparticles.

### 3.5. Nanoparticle Release

There are major concerns about the likelihood of nanoparticles leaching from polymeric membrane matrices during filtration and cleaning [53], as the leaching of nanoparticles would result in secondary pollution. Therefore, the leaching of Ag and Pd nanoparticles was investigated. No Ag or Pd was detected in the feed, permeate, or concentrate samples for the investigated duration. This showed the stability of the additives in the membrane matrix. However, it is also possible that Ag and Pd were not detected because leaching experiments were conducted for a short period (12 h). The leaching of nanoparticles also occurs during membrane cleaning, especially when polymeric membranes are subjected to harsh cleaning conditions [53]. Therefore, long-term filtration experiments, as well as the effects of membrane cleaning on nanoparticle leaching, need further investigation.

## 4. Conclusions

In this study, it was found that the in situ generation of Ag/Pd nanoparticles in PES membranes enhanced the physical and filtration properties of the fabricated membranes. The Ag/Pd PES entrapped membranes were more hydrophilic and showed higher fluxes compared to pristine PES membrane. The entrapment of Ag/Pd nanoparticles did not improve salt rejection. However, low salt rejection is beneficial in wastewater treatment, where the feed contains low salt concentration. In addition, the membranes suffered less flux decline due to concentration polarization (CP), which improves the energy efficiency of the membranes as CP decreases the driving force, leading to flux decline. Increasing the applied pressure (therefore energy) becomes necessary to maintain high fluxes. Ag/Pd PES membranes can be applied for the treatment of water from various sources contaminated with EOCs. However, the quality of the treated water may not be suitable for drinking, but can could nonetheless be used for other purposes, as the organics are not completely removed by these membranes. Ag/Pd PES membranes could thus be used for the pre-treatment of industrial water containing pharmaceutical organics.

## Figures and Tables

**Table 1:** Composition of the PES casting solutions (wt%). [Ag/Pd is the combined ratio of Ag and Pd nanoparticles].

Membrane	Concentration (wt%)				
	PES	TEP	Ag	Pd	Ag/Pd
M0	15	85.00	0	0	0
M1	15	84.90	0.05	0.05	0.10
M2	15	84.85	0.075	0.075	0.15
M3	15	84.80	0.10	0.10	0.20
M4	15	84.75	0.125	0.125	0.25

**Table 2:** Physical properties of selected organic compounds.

Compound	MW (g·mol^−1^)	Charge (pH 7)	Log K_ow_
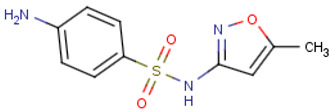 Sulfamethoxazole (C_10_H_11_N_3_O_3_S)	253.278	Negative	0.791
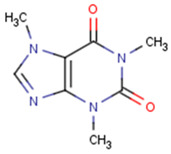 Caffeine (C_8_H_10_N_4_O_2_)	194.1906	Neutral	−0.546
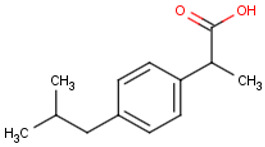 Ibuprofen (C_13_H_18_O_2_)	206.2808	Negative	3.844
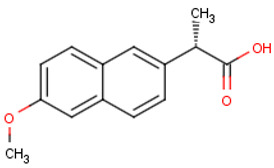 Naproxen (C_14_H_14_O_3_)	230.2592	Negative	2.986
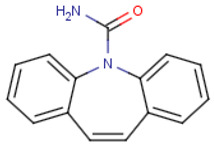 Carbamazepine (C_15_H_12_N_2_O)	236.2686	Neutral	2.766

**Table 3:** Membrane properties: bulk porosity, contact angle, pure water permeability (Lp), zeta potential, mean pore radius and salt rejection.

Membrane	Bulk Porosity (%)	Contact Angle (°)	Lp (Lm^−2^h^−1^bar^−1^)	Zeta Potential	Mean Pore Radius (nm)	Salt Rejection (%)	
						NaCl	MgSO_4_
M0	57 ± 1.1	72.6 ± 3.6	37 ± 1.5	−42.48 ± 0.48	2.39	2.3 ± 0.2	10.5 ± 1.2
M1	59 ± 0.4	71.0 ± 3.5	39 ± 4.4	−38.21 ± 0.41	2.59	4.1 ± 0.5	12.6 ± 0.8
M2	62 ± 0.9	64.7 ± 3.2	59 ± 4.3	−34.36 ± 0.36	3.74	3.8 ± 0.8	14.8 ± 1.1
M3	68 ± 0.8	61.2 ± 3.1	75 ± 8.1	−31.29 ± 0.34	4.68	1.4 ± 0.3	9.4 ± 0.9
M4	77 ± 1.2	51.3 ± 2.7	85 ± 1.7	−31.29 ± 0.52	4.70	4.6 ± 0.6	13.4 ± 1.3

## Abbreviations

AFM	Atomic Force Microscopy
BSA	Bovine serum albumin
CapEX	Capital expenditure
CP	Concentration polarization
EDCs	Endocrine-disrupting compounds
EDS	Energy Dispersive X-ray Spectroscopy
EOCs	Emerging Organic Compounds
FRR	Flux recovery ratio
FTIR	Fourier Transform Infrared
GO	Graphene oxide
HRTEM	High resolution transmission electron microscopy
ICP-MS	Inductive coupled plasma mass spectroscopy
L_p_	Pure water permeability
MSPs	Membrane separation processes
Mw	Molecular weight
MWCO	Molecular weight cut-off
NF	Nanofiltration
NTU	Nephelometric turbidity unit
OpEX	Operating expenditure
PES	Polyethersulfone
PSf	Polysulfone
PVDF	Polyvinylidene fluoride
R_ir_	Irreversible fouling ratio
RO	Reverse osmosis
R_r_	Reversible fouling ratio
R_t_	Total fouling ratio
S_a_	Average arithmetic roughness
SEM	Scanning Electron Microscopy
S_q_	Mean square roughness
TEM	Transmission Electron Microscopy
TEP	Triethyl phosphate
TGA	Thermogravimetric analysis
TMP	Trans-membrane pressure
TOC	Total organic carbon
UF	Ultrafiltration
